# A Two-Stage Contrastive Learning Framework Grounded in Label-Specific Features for Low-Frequency Labels in Chest X-Ray Multi-Label Classification

**DOI:** 10.3390/bioengineering13050553

**Published:** 2026-05-13

**Authors:** Shi Tang, Meiyan Huang, Qianjin Feng

**Affiliations:** 1 School of Biomedical Engineering, Southern Medical University, Guangzhou 510515, China; 2 Guangdong Provincial Key Laboratory of Medical Image Processing, Southern Medical University, Guangzhou 510515, China; 3 Guangdong Province Engineering Laboratory for Medical Imaging and Diagnostic Technology, Southern Medical University, Guangzhou 510515, China

**Keywords:** image analysis and processing, computer-aided diagnosis, multi-label disease classification on chest X-rays

## Abstract

Thoracic diseases represent a significant threat to human health. Chest X-ray imaging, owing to its cost-effectiveness and rapid imaging capabilities, has been widely adopted as a primary diagnostic tool in clinical practice. However, existing models are often susceptible to imbalances in disease label distributions. This study proposes a dual-phase convolutional neural network for the classification of thoracic diseases. In the first phase, matrix operations are employed to extract discriminative features corresponding to each disease label, effectively shifting the classification task from the image domain to a label-specific feature domain. The second phase incorporates feature contrastive loss and feature updating mechanisms to further enhance the model’s generalization capability. The proposed framework was evaluated on three public datasets (CheXpert, REFLACX, and EGD) to verify its consistent performance across diverse data sources. Experimental results demonstrate that our model achieved an AUC of 0.8296, AUPRC of 0.2969, Precision of 0.3943, and F1-score of 0.3301 on our dataset, outperforming existing chest X-ray classification models. These findings indicate that our proposed framework effectively learns label-specific characteristics and captures intrinsic image features associated with each disease label, offering an advanced technical tool for the diagnosis of thoracic diseases.

## 1. Introduction

The rapid advancement of deep learning and predictive modeling has driven substantial progress across a wide range of scientific disciplines [1,2]. Building upon these broad technological foundations, computer vision has achieved remarkable success in medical imaging applications [3,4]. This progress is particularly promising in pulmonary medicine, where chest diseases remain among the leading causes of global morbidity and mortality. Chest radiography, owing to its cost-effectiveness, low radiation exposure, and rapid imaging capability, serves as the primary and most widely used diagnostic tool for thoracic pathologies, including pneumonia, pulmonary edema, pleural effusion, and lung malignancies [5]. However, current clinical practice faces two critical challenges: (1) substantial inter-observer variability, often caused by differences in expertise or subjective interpretation; (2) high diagnostic workload, particularly in busy settings such as emergency departments or large-scale screening programs [6]. To mitigate these issues, researchers have increasingly adopted computer-aided diagnosis (CAD) systems powered by deep learning, aiming to improve both the efficiency and accuracy of image interpretation and to facilitate subsequent treatment planning [7].

A primary bottleneck hindering the advancement of CXR-based CAD systems is the prevalence of multi-label co-occurrence and long-tail distributions in real-world clinical scenarios. Clinically, conditions such as pneumonia, emphysema, and cardiomegaly frequently co-occur; meanwhile, the severe imbalance in sample sizes across different categories in training datasets leads to a substantially poorer performance in identifying rare diseases or early-stage subtle lesions compared to common pathologies. Existing studies have attempted to address these challenges from both data perspectives like resampling, GAN-based augmentation and model training perspective like weighted loss functions, transfer learning, attention mechanisms [8,9,10]. However, while these methods alleviate the pressure of class imbalance, most current models still extract image features holistically, mapping the entire radiograph to a single global feature vector. This paradigm fails to capture and utilize the distinct image representations specific to each disease label. Given that the underlying logic of multi-label classification resides in the precise capture and validation of positive features for independent diseases, this study proposes a novel approach that shifts from global image representation to explicit, label-wise feature modeling. We design a two-stage framework: the first stage leverages large-scale public datasets to extract and store label-specific feature vectors in a structured feature pool; the second stage introduces similarity constraints and a dynamic update mechanism to iteratively refine these features, ensuring the model focuses on robust, disease-specific characteristics and effectively mitigates label imbalance from a feature representation perspective. The main contributions of this study are asfollows:

1. A label-aware pre-training strategy featuring a novel Label-Specific Feature Extractor (LSFE) is designed. By creatively utilizing classification weights for pixel-wise semantic projection, this strategy fundamentally transforms multi-label classification from global image-level representation into independent, label-wise feature modeling.
2. A structured feature bank tailored for low-frequency labels is introduced. Furthermore, explicit feature-level augmentation is implemented to directly address severe class imbalance within the feature space.
3. A novel loss function composed of align- and margin-contrastive loss terms is proposed to guide feature alignment with the feature bank, ensuring compactness within classes and dispersion across classes for each label.
4. A dynamic feature pool update mechanism is incorporated during the fine-tuning stage. By iteratively replacing outdated source-domain features with newly extracted target-domain representations, this mechanism explicitly aligns the feature bank with the target data distribution, effectively mitigating domain shift and improving the model’s transferability across different clinical datasets.

## 2. Related Works

### 2.1. Multi-Label Thoracic Disease Classification

According to the Forum of International Respiratory Societies (FIRS), chronic respiratory conditions—such as asthma alone—affect over 3 million people worldwide [11], underscoring the urgent need for efficient, AI-assisted diagnosis of thoracic and pulmonary disorders. Against this backdrop, employing deep learning techniques to perform disease classification based on chest X-rays (CXR) holds significant practical value, as it can assist clinicians in making more accurate and timely treatment decisions.

Existing studies primarily focus on optimizing feature extraction capabilities through advanced deep learning architectures. Early approaches were based on convolutional neural networks (CNNs): for instance, Rajpurkar et al. [12] proposed CheXNet, a 121-layer DenseNet model that achieved classification of 14 thoracic diseases on the CheXpert dataset, which for the first time validated the potential of deep learning in multi-label medical imaging tasks. Building on CNN advancements, researchers have developed architecture-specific optimizations targeting chest X-ray characteristics: a study by Zhao and Wang [13] proposed a CNN architecture that integrates large-kernel convolutions with anatomical segmentation, employing a four-stage feature extraction network to capture global contextual information. ChestNet [14], proposed by Wang et al., incorporates an attention branch in parallel with the classification branch, allowing the network to automatically focus on lesion regions and thereby improve classification accuracy across 14 thoracic disease categories. Subsequent studies introduced Transformer architectures to address the limitations of CNNs in modeling long-range lesion correlations. The Med-former [15] introduces a local-global Transformer module, which concurrently captures global contextual information and local fine-grained features, thereby minimizing information loss and enhancing the representation of critical regions such as lesion areas. Ma et al. [16] constructed an open foundation model based on Swin Transformer Large, cyclically training and integrating it across multiple public datasets. The subsequently appended multi-task head enables the execution of diverse downstream tasks, including classification, segmentation, and report generation. Yu and Zhou [17] improved the DETR (Detection Transformer) framework by incorporating Efficient Channel Attention (ECA-Net) and Spatial Attention Upsampling (SAU) modules, significantly strengthening feature representation for small lesions. Beyond pure classification architectures, hybrid frameworks combining segmentation and feature extraction have shown promise. For example, Study [18] utilizes a specifically designed Deep Atrous Attention U-Net (DAA-UNet) for lung segmentation, optimizing the Region of Interest to improve classification performance. Classification is subsequently performed using transfer learning with fine-tuned pre-trained models on the segmented lung regions. Ahmad et al. [19] developed a novel collaborative framework combining GVit and Swin Transformer architectures, which demonstrated high accuracy in chest disease detection. In addition, several studies have focused on modeling label dependencies or enhancing rare samples to improve model diagnostic performance [7,20,21].

Nevertheless, existing multi-label classification methods generally suffer from poor adaptability to small datasets and insufficient integration of local pathological details. Most models rely on large-scale, balanced datasets for training. When transferred to local small datasets (e.g., single-center clinical data), significant performance degradation occurs due to feature distribution shifts caused by differences in imaging equipment or patient populations [22].

### 2.2. Pre-Training and Knowledge Transfer in Medical Imaging

To alleviate the insufficiency of feature learning in small datasets, knowledge transfer based on large-scale data has become a mainstream approach in medical imaging. Its core idea is to transfer “general feature extraction capabilities” learned from large-scale datasets to small-dataset tasks.

Existing transfer strategies can be broadly categorized into two paradigms. The first involves transfer from general-domain to medical-domain data [12,23]. However, pre-training with natural images exhibits inherent limitations. The primary drawback stems from the significant domain shift between natural images and medical images, as the statistical characteristics and feature distributions differ substantially. The second transfer strategy, which has gained wider adoption in current research, involves transfer learning from large-scale medical datasets to small-scale medical datasets. AbdomenAtlas [24] is pre-trained on abdominal CT datasets and then transferred to small-scale tasks such as liver tumor segmentation. Through domain-adaptive fine-tuning, it achieves improved performance. Xie et al. [25] utilize a large amount of 2D chest X-rays and 3D CT data to pre-train a Transformer model via self-supervised learning. When transferred to the field of 3D lung cancer segmentation, the model delivers favorable performance.

However, existing transfer methods suffer from coarse transfer granularity and insufficient adaptation to label-specific characteristics. Most methods only transfer model parameters and fail to extract exclusive, reusable label-specific features for individual diseases. This lack of “label-level precise knowledge transfer” makes it difficult for models to effectively learn features of rare labels in small datasets. This study addresses this gap by extracting label-specific features from large-scale datasets via matrix operations, which supplements and optimizes label-level knowledge transfer. Unlike existing graph-based correlations or standard instance-level contrastive learning, our approach avoids complex graph construction and global feature homogenization. Instead, we establish a novel transfer learning paradigm. By dynamically updating a label-specific feature bank and applying tailored align- and margin-class contrastive constraints, our framework explicitly isolates and enhances the representation of low-frequency diseases, filling a significant void in current CAD systems.

## 3. Methods

**Figure 1:**
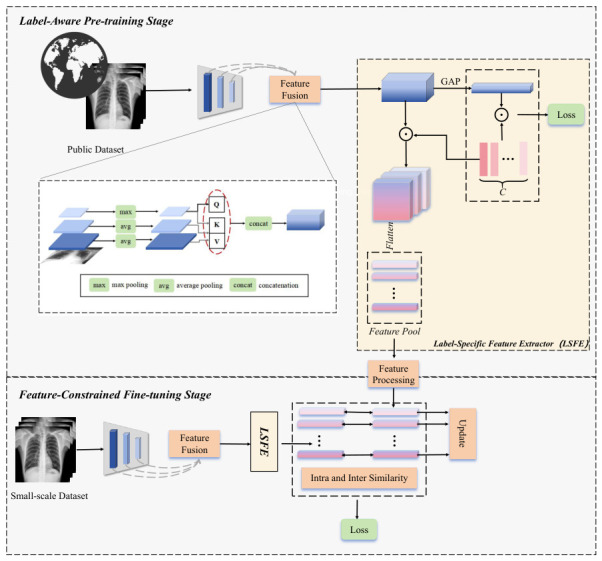
The proposed method consists of two main stages: the label-aware pre-training stage and the feature-constrained fine-tuning stage. (1) The pre-training stage learns class-specific representative features for each disease label from a large-scale public dataset. These features are then processed through a feature processing module and stored in a feature pool structure. (2) The fine-tuning stage is fine-tuned using a small-scale dataset, where the extracted features are compared for similarity both within and outside the feature pool. The features in the feature pool are then updated accordingly.

The overall framework of our proposed method is illustrated in Figure 1. As shown, it consists of two key stages: the label-aware pre-training stage and the feature-constrained fine-tuning stage, with the Label-Specific Feature Extractor serving as the core component in both. The label-specific representations obtained through the feature extractor during the pre-training stage on a large-scale dataset are stored in a structured feature pool. In the adaptive fine-tuning model, newly extracted features are constrained by comparison with the stored representations in the feature pool. Through a dynamic update-and-replacement mechanism, less representative vectors in the pool are progressively eliminated, enabling the model to retain and refine a set of highly discriminative, label-specific feature vectors. In the following sections, we elaborate on the specific roles and implementation details of each component.

### 3.1. Label-Aware Pre-Training Stage

During the pre-training phase, the model takes as input preprocessed images resized to 256×256 pixels. Given an input image *I*, it is associated with a set of labels $Y=\left\{y_{1},y_{2},\ldots ,y_{N}\right\}$, where $y_{i}\in \left\{0,1\right\}$. Here, $y_{i}=1$ indicates that the medical image is positive for the $i_{th}$ label, *N* indicates the number of the labels, and the physician needs to formulate an appropriate treatment strategy for the corresponding disease.

#### 3.1.1. Feature Fusion

The input images are fed into a backbone neural network, where successive convolutional layers extract hierarchical features. These features are subsequently transformed into classification outputs by a final classification layer. Since convolutional layers at different depths capture features at varying semantic levels—shallower layers encoding structural details and deeper layers encoding abstract semantics—it is essential to ensure that the extracted representations preserve both types of information. Inspired by the AFPF structure proposed in [26], the pre-training model performs feature fusion using the outputs of the last three convolutional layers of the backbone. Feature maps from the last three convolutional layers of the baseline convolutional network are first extracted. Prior to performing self-attention and cross-layer attention operations, each feature map undergoes downsampling via either max pooling or average pooling. Following the attention operations, all resulting feature maps are concatenated to form the final, fully fused feature representation.

The fused feature X corresponding to the input can be obtained after the convolutional operations of the baseline network and the feature fusion module, and is given as follows:


(1) $$\begin{array}{cc} X=\mathrm{FeatFuse}( & \max\left(\sigma_{i}\left(I\right)\right),\mathrm{avg}\left(\sigma_{i-1}\left(I\right)\right),\mathrm{avg}\left(\sigma_{i-2}\left(I\right)\right)) \end{array}$$

In this context, FeatFuse(·) denotes the feature fusion operation, and σ(·) represents the convolution operation. Assuming that the backbone network consists of *i* convolutional layers, the fusion process selects the feature maps from the final three layers for aggregation. During feature fusion, max(·) and avg(·) refer to max pooling and average pooling operations, respectively. These operations assist the network in reducing spatial dimensionality while preserving important features and enhancing robustness, without discarding any feature channels or representative information. Additionally, applying attention mechanisms across features from different layers enables the fused representation to capture richer semantic and structural information.After global average pooling is applied to the fused features, the model produces the final classification results through a classification head and is trained using a classification loss.

#### 3.1.2. Label-Specific Feature Extractor

In parallel with the classification branch, and drawing inspiration from [7], this study proposes a Label-Specific Feature Extractor (LSFE) structure that innovatively introduces the extraction of label-specific representation vectors into our designed network. Unlike conventional pooling methods, we leverage the semantic knowledge embedded in the classification head $W_{clf}\in R^{C\times N}$. Because these weights are optimized over a large-scale and diverse dataset during pre-training, they are prevented from degenerating into narrow decision-boundary artifacts. Instead, we treat each column of $W_{clf}$ as a robust, category-specific learnable prototype that encapsulates the core visual attributes of the *i*-th pathology. Formally, given the fused feature map $X\in R^{C\times H\times W}$, we perform a pixel-wise semantic projection. By computing the outer product between the feature channels and the classification weights via Einstein summation, we derive a set of high-dimensional tensors F:


(2) $$F=\mathrm{einsum}{(}^{\prime }chw,cn\to nchw^{\prime },X,W_{clf})$$

This operation can be interpreted as modulating the global feature map with class-specific importance weights. Each slice $F_{i}\in R^{C\times H\times W}$ effectively filters out irrelevant background noise and retains features highly correlated with the *i*-th label. Subsequently, to obtain a compact representation for the feature bank, we apply a flatten operation:


(3) $$Z_{i}=\mathrm{Flatten}\left(F_{i}\right)\in R^{C\times (H\times W)}$$

This mechanism ensures that $Z_{i}$ is spatially and semantically grounded in the *i*-th category, providing a more stable and discriminative basis for the subsequent contrastive learning stages compared to traditional global pooling. Therefore, the detailed training process of this stage can be summarized and presented as illustrated in Algorithm 1. Specifically, to prevent the feature bank from being contaminated by model misclassifications, we implement a strict entry criterion during the initialization stage. As shown, only feature vectors from samples that are correctly classified are admitted into the pool. This ensures that the stored prototypes represent true pathological characteristics rather than erroneous model artifacts.
**Algorithm 1** Stage 1: Label-Aware Pre-training and Initialization**Require:** 
Pre-trained backbone $f_{\theta }$, training set $D_{pre}$, total labels *N*.**Ensure:** 
Partially optimized θ, initialized feature bank P.  1:**for** each epoch in Stage 1 **do**  2:    **for** batch $\left(I,Y\right)\in D_{pre}$ **do**  3:        Extract fused features $X=\mathrm{FeatFuse}(f_{\theta }\left(I\right))$  4:        Compute multi-label classification loss $L_{cls}$  5:        Update θ by minimizing $L_{cls}$  6:    **end for**  7:**end for**  8:// *Feature Bank Initialization*  9:P←∅10:**for** each sample *I* in $D_{pre}$ **do**11:    **if** *I* is correctly classified for label *i* **then**12:        Extract $Z_{i}$ and store into $P_{i}=\left\{P_{i}^{+},P_{i}^{-}\right\}$13:    **end if**14:**end for**

### 3.2. Feature Pool

The feature pool is a structure proposed to store discriminative feature vectors. For each label, both its positive and negative representative features are stored in a feature pool of size Num×d, where Num denotes the number of representative vectors per label, and d represents the dimensionality of each vector. With these label-specific representative vectors stored in the feature pool, features extracted from new samples can be compared against the stored representations, allowing the model to capture more discriminative features from images for downstream classification tasks.

**Figure 2:**
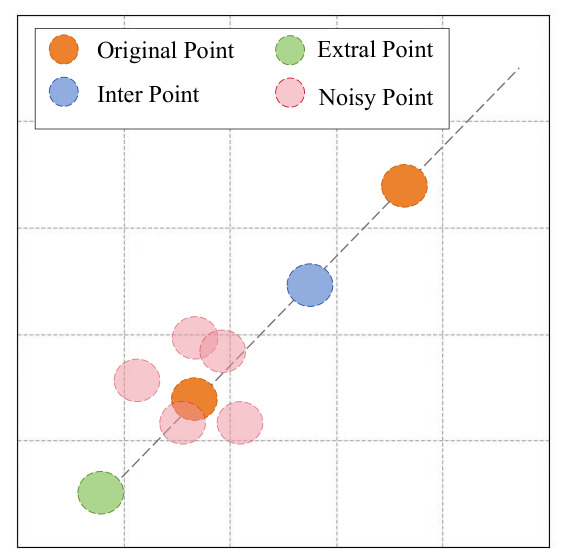
Two-dimensional spatial schematic diagram of the three techniques adopted in the augmentation of positive vectors: interpolation, extrapolation, and random noise.

Due to the imbalance between positive and negative samples for certain labels, the number of feature vectors stored in the positive sample pool may be significantly smaller than that in the negative pool. To mitigate the impact of such imbalances on subsequent model performance, and inspired by the data augmentation strategy proposed by Taylor et al. [27], we apply three techniques—interpolation, extrapolation, and random noise—to expand the set of positive vectors. Conceptually, if feature vectors are regarded as points in a two-dimensional plane, interpolation generates new vectors along the line segments connecting pairs of existing points, while extrapolation produces new vectors along the extension of these lines. Random noise vectors are generated by applying stochastic perturbations to the original features. The specific reference method is shown in Figure 2.

Additionally, for high-frequency labels (both positive and negative), we perform clustering using the K-Means algorithm to extract the most representative and discriminative features while reducing computational overhead. This process yields Num cluster centroids per label, which are retained as the representative feature set in the feature pool.

Through feature augmentation and clustering within the feature pool, a sufficient number of representative feature vectors is obtained for each label, which can subsequently be utilized for contrastive learning.

### 3.3. Feature-Constrained Fine-Tuning Stage

In this part, we refine the classification model obtained from the pre-training module by using samples from the feature pool and comparing the label-specific features extracted by the model. The specific model details are as follows:

#### 3.3.1. Supervised by Contrastive Loss Constraints

By fine-tuning the pre-training model with a small batch of new data, the feature extractor can generate new feature vectors that reside in the same feature space as those stored in the feature bank.

Assume the label of the input image I can be described as $Y=\left\{y_{1},y_{2},\ldots ,y_{N}\right\}$. If $y_{i}=1$, then the label-specific feature extracted by the model $Z_{i}\in R^{d}$, should be closer to the $i_{th}$ label feature in the positive pool than to that in the negative pool. Conversely, if $y_{i}=0$, then $Z_{i}$ should be closer to the $i_{th}$ label feature in the negative pool.

In this study, we use the average cosine similarity between feature vectors to quantify the distance between the extracted features and those stored in the feature pool. To address the potential ambiguity caused by label-specific features lying in the overlapping region between the positive and negative pools which may result in classification confusion, we introduce an additional comparative mechanism beyond average similarity measurements. Specifically, we compute both theoretical similarity and cross-pool similarity, which serve to reduce the influence of confounding features from the opposing pool.

**Figure 3:**
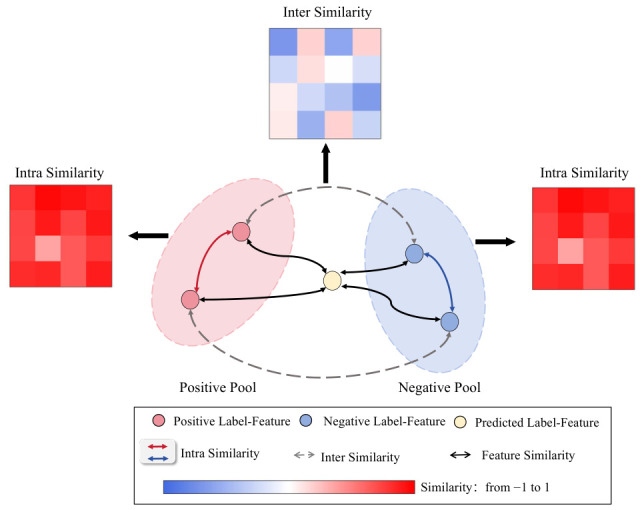
Computational Schematic of align- and margin-Similarity Loss. Feature constraints are implemented by leveraging the intra-pool and inter-pool similarity derived from feature pooling.

The detailed implementation procedure is illustrated in Figure 3. Let $D_{neg}$ denote the similarity between the label-specific feature vector of a new sample and the corresponding negative vectors stored in the feature pool. Then, $D_{neg}$ is computed as:


(4) $$\begin{array}{c} D_{\mathrm{neg}}=\frac{1}{N}\sum_{i=1}^{N}Sim_{\cos}\left(f_{\mathrm{ext}},f_{\mathrm{neg}\_i}\right) \end{array}$$

Here, $Sim_{\cos}$ denotes the cosine similarity function, $f_{\mathrm{ext}}$ represents the newly extracted label-specific feature vector, and $f_{\mathrm{neg}\_i}$ refers to the stored vectors in the negative label feature pool. Analogous to the definition of $D_{\mathrm{neg}}$, the similarity metric between the novel features and the stored positive features, denoted as $D_{\mathrm{pos}}$, is defined as:


(5) $$\begin{array}{c} D_{\mathrm{pos}}=\frac{1}{N}\sum_{i=1}^{N}Sim_{\cos}\left(f_{\mathrm{ext}},f_{\mathrm{pos}\_i}\right) \end{array}$$

To further encourage the extracted features to align with the correct feature pool while minimizing the overlap between positive and negative representations, we introduce a cross-similarity supervision term, denoted as $D_{\mathrm{cross}}$, which measures the similarity between the positive and negative feature pools of the same label, and is defined as:


(6) $$\begin{array}{c} D_{\mathrm{cross}}=\frac{1}{N^{2}}\sum_{i=1,j=1}^{N}Sim_{\cos}\left(f_{\mathrm{pos}\_i},f_{\mathrm{neg}\_j}\right) \end{array}$$

(7) $$\begin{array}{cc} L_{\mathrm{align}}= & \;y_{i}\left[\mathrm{ReLU}\left(D_{\mathrm{neg}}-D_{\mathrm{pos}}\right)\right]\\ \; & +\left(1-y_{i}\right)\left[\mathrm{ReLU}\left(D_{\mathrm{pos}}-D_{\mathrm{neg}}\right)\right] \end{array}$$

(8) $$\begin{array}{cc} L_{\mathrm{margin}}= & \;y_{i}\left[\mathrm{ReLU}\left(D_{\mathrm{neg}}-D_{\mathrm{cross}}\right)\right]\\ \; & +\left(1-y_{i}\right)\left[\mathrm{ReLU}\left(D_{\mathrm{pos}}-D_{\mathrm{cross}}\right)\right] \end{array}$$

Integrating the classification loss $L_{\mathrm{cls}}$ from the downstream task, the final objective function $L_{\mathrm{final}}$ for model training is formulated as:


(9) $$\begin{array}{c} L_{\mathrm{final}}=L_{\mathrm{cls}}+L_{\mathrm{align}}+L_{\mathrm{margin}} \end{array}$$

#### 3.3.2. Update to the Feature Pool

To enhance the adaptability of features stored in the feature bank to the novel, small-scale dataset used in the main model, the authors introduce an additional feature pool update and elimination mechanism during training.

**Figure 4:**
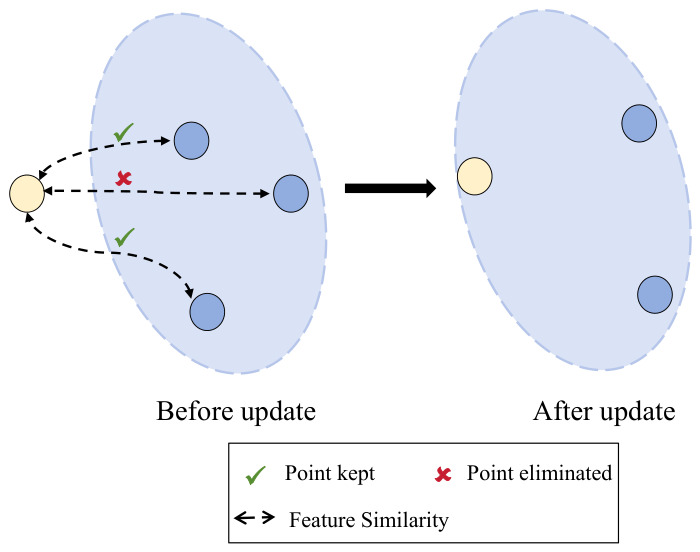
Demonstration of the Feature Pool Update Process. In this diagram, the yellow circle represents the newly extracted label-specific feature from the current sample, while the blue circles denote the existing representation vectors stored in the feature pool. During each update, the number of added and removed feature vectors is kept equal in order to maintain a constant storage capacity within the feature pool.

As illustrated in Figure 4, the similarity between features can be intuitively visualized as the Euclidean distance between corresponding points in a two-dimensional space, where higher similarity is reflected by a shorter distance between the feature points. When an image is correctly classified with the $i_{th}$ label, its corresponding label-specific feature vector is added to the feature pool. Subsequently, the vector exhibiting the lowest similarity to the newly added feature is removed from the pool, thereby maintaining a constant number of vectors within the feature bank. This update operation is performed at each epoch. Importantly, this process explicitly involves discarding outdated instance prototypes from the memory bank to accommodate new representations extracted from the target domain. Consequently, this update functions as a dynamic domain-alignment strategy rather than a traditional dimensionality reduction technique, ensuring that the contrastive reference pool smoothly adapts to the local clinical data distribution.

Through the proposed training paradigm, we achieve the effective extraction, storage, and timely updating of label-specific features. To provide a more intuitive understanding of the training procedure, the comprehensive workflow is detailed in the following Algorithm 2.
**Algorithm 2** Stage 2: Feature-Constrained Fine-tuning and Dynamic Update**Require:** 
Optimized θ from Stage 1, LSFE module $g_{\phi }$, Feature bank P, learning rate η.**Ensure:** 
Fully optimized parameters θ,ϕ.  1:**for** each epoch in Stage 2 **do**  2:   **for** batch $\left(I,Y\right)\in D_{fine}$ **do**  3:        $X=\mathrm{FeatFuse}(f_{\theta }\left(I\right))$  4:        **for** each label i=1…N **do**  5:           // *LSFE Feature Extraction*  6:           $Z_{i}=\mathrm{Flatten}\left(\mathrm{einsum}{(}^{\prime }chw,cn\to nchw^{\prime },X,W_{clf})\right)$  7:           // *Contrastive Loss Calculation*  8:           Compute $D_{\mathrm{pos}},D_{\mathrm{neg}}$ using $P_{i}^{+},P_{i}^{-}$  9:           $L_{\mathrm{align}}=Y_{i}ReLU\left(D_{\mathrm{neg}}-D_{\mathrm{pos}}\right)+\left(1-Y_{i}\right)ReLU\left(D_{\mathrm{pos}}-D_{\mathrm{neg}}\right)$10:           $L_{margin}=Y_{i}ReLU\left(D_{\mathrm{neg}}-D_{\mathrm{cross}}\right)+\left(1-Y_{i}\right)ReLU\left(D_{\mathrm{pos}}-D_{\mathrm{cross}}\right)$11:        **end for**12:        $L_{\mathrm{final}}=L_{\mathrm{cls}}+\sum \left(L_{\mathrm{algin}}+L_{\mathrm{margin}}\right)$13:        Update θ,ϕ via backpropagation14:        // *Dynamic Update of P*15:        **if** *I* is correctly classified for label *i* **then**16:           Update $P_{i}$ by adding $Z_{i}$ and removing least similar vector17:        **end if**18:   **end for**19:**end for**

## 4. Experiment

### 4.1. Dataset

**Figure 5:**
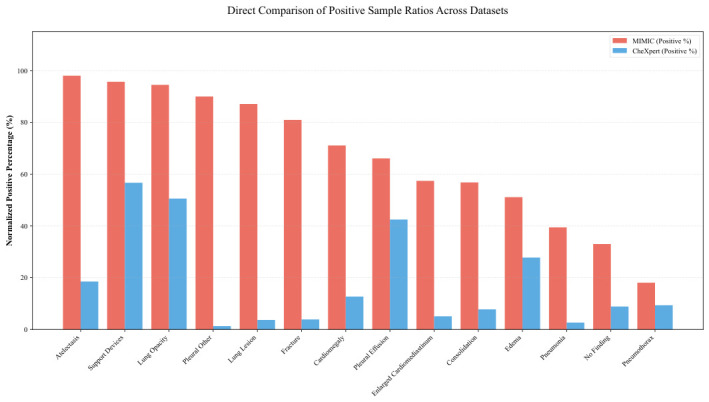
Proportion of positive, negative, and uncertain samples for each label in the CheXpert and MIMIC datasets.

Three datasets—CheXpert [28], REFLACX [29], and EGD [30]—were used in this study. CheXpert is a large-scale public medical image dataset released by Stanford University, consisting of 224,316 chest radiographs collected from 65,240 patients. Due to computational limitations, only a subset of posteroanterior (PA) chest radiographs was selected for training the pre-training model. The REFLACX dataset comprises 2616 chest X-ray images, while the EGD contains a total of 1083 public chest X-ray (CXR) images. The REFLACX and EGD datasets were both derived from the MIMIC-CXR database [31], a large-scale publicly available chest radiograph dataset collected from the Beth Israel Deaconess Medical Center. To verify the label distribution difference between the two large-scale dataset, we generated a bar plot depicting the positive/negative sample ratios for all labels as shown in Figure 5.

### 4.2. Implementation Details

During both the pre-training and main model training phases, all chest radiographs were uniformly resized to 256×256 pixels through standardized preprocessing procedures. ResNet50 was adopted as the backbone network, with CheXpert serving as the source dataset for the pre-training phase. Given limited computational resources, only posteroanterior view radiographs were retained from the CheXpert dataset.

Following pre-training, the model produced a collection of label-specific feature vector pools, each constrained to a maximum size of 300 vectors. During the fine-tuning phase of the model, we utilized the REFLACX dataset for training and the EGD dataset for validation. Five-fold cross-validation was employed, and the average performance across all folds was reported. All experiments were conducted on two NVIDIA GeForce RTX 3090 GPUs (NVIDIA Corp., Santa Clara, CA, USA) using the PyTorch (version 1.13.0) framework. The Adam optimizer was applied with parameters $\beta_{1}=0.9$ and $\beta_{2}=0.999$, a weight decay set to 0.0001, an initial learning rate of 0.001, and a batch size of 40.

### 4.3. Results Analysis

#### 4.3.1. Comparison with State-of-the-Art Models

To validate the effectiveness of the proposed method, we compare it against several state-of-the-art baseline models, including CheXNet [12], SwinCheX [32], CVTGNet [33], MLGCN [34], DANN [35], SimCLR [36], Class-Center Guider [37], and ResNet50 models pre-trained on ImageNet [38] and CheXpert datasets, respectively.

CheXNet and SwinCheX are models pre-trained on large-scale chest X-ray datasets using DenseNet and Swin Transformer architectures, respectively. In contrast, CVTGNet and MLGCN follow a research direction that incorporates graph convolutional modules into networks, aiming to leverage inter-label co-occurrence relationships to facilitate model training. DANN, SimCLR, and Class-Center Guider represent research methodologies involving knowledge transfer. It is worth noting that the baselines selected for comparison, such as SimCLR and Class-Center Guider, were chosen because like our method, they focus on representation learning, prototype alignment, and knowledge transfer within deep neural networks. Because our dynamic feature pool update replaces instance prototypes rather than reducing feature dimensionality, classical dimensionality reduction or feature selection algorithms (e.g., mRMR [39], ReliefF [40]) are architecturally incompatible with our end-to-end contrastive framework and thus were not included in the baseline comparisons.

All baseline methods, along with the proposed approach, were evaluated under identical experimental settings using the same dataset. The comparative results against other state-of-the-art methods are presented in Table 1. In this study, model performance is primarily evaluated using five metrics: AUC, AUPRC, F1-score, Precision, and Recall. Notably, to better reflect the independent performance of each label and ensure fair assessment of the model’s capability on rare disease categories, the metrics are calculated using the macro-averaging strategy.

As shown, the network architecture proposed in this study outperforms all compared methods in terms of AUC, AUPRC, F1-Score, Precision, and Recall, achieved an AUC of 0.8296, an AUPRC of 0.2969, an F1-score of 0.3301, a precision of 0.3943, and a recall of 0.2839, respectively, which thus confirms the effectiveness of our approach in maintaining a balanced trade-off between precision and recall.

#### 4.3.2. Comparison Under Specific Disease Labels

Class imbalance, particularly poor classification performance on rare disease labels, has long posed a major challenge in multi-label medical image classification. To assess whether the proposed method offers improved performance over conventional approaches at the individual label level, we selected six disease labels with relatively low positive sample ratios from the original 14-label dataset. A comprehensive comparison was conducted between our method and baseline models using five evaluation metrics: area under the curve (AUC), area under the ROC curve (AUC), Precision, Recall, and F1-Score.

**Figure 6:**
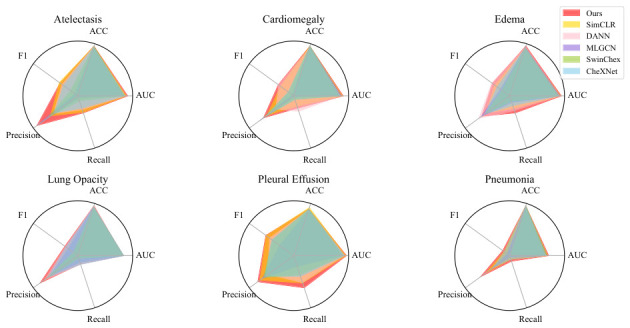
Performance comparison across specific disease labels. The red polygon in the figure represents the proposed method in this study.

For all six selected labels, the proportion of positive samples in the training set remained below 25%. Notably, the “Edema” label had a positive sample ratio close to 10%, while “Pneumothorax” exhibited a more severe imbalance, with fewer than 5% positive samples. The comparative results are visualized in the radar plots shown in Figure 6.

In each subplot, the red polygon represents the performance of the proposed model across the five evaluation metrics. As illustrated, our model consistently outperforms the baseline methods across multiple disease labels and metrics. These findings indicate that the proposed contrastive learning strategy—based on inter- and intra-feature pooling—effectively captures label-specific discriminative features and enhances classification accuracy, particularly for rare diseases. This improvement suggests that our approach holds strong potential as a robust diagnostic aid in clinical settings, especially for underrepresented conditions.

#### 4.3.3. Bidirectional Transfer and Validation

To verify that our proposed method exhibits excellent generalization performance across different datasets, we designed Bidirectional Transfer experiments. The specific experimental setup is as follows: During the Feature-Constrained Fine-Tuning Stage, we utilized two datasets, REFLACX and EGD, and constructed three groups of comparative experiments. Specifically, the first group uses the REFLACX dataset for fine-tuning and the EGD dataset for validation; the second group employs the EGD dataset for fine-tuning and the REFLACX dataset for validation; the third group combines the two datasets, using the merged dataset for both fine-tuning and validation. Notably, to avoid the impact of differences in dataset sizes on the experimental results, instead of using all samples from the REFLACX dataset, we extracted a subset of samples from REFLACX with the same sample size as EGD, while maintaining the positive sample ratio of each label as constant as possible. The final comparative experimental results are as Table 2. As can be seen from our comparison results, our proposed scheme has achieved favorable model performance across all fine-tuning and validation dataset partitioning schemes. The consistent performance observed in Table 2 serves as strong empirical evidence that the label-specific features extracted by the LSFE encode stable pathological semantics rather than source-domain artifacts. If the classifier weights were merely overfitted boundaries, severe performance degradation would occur during cross-dataset transfer. This also demonstrates that our scheme can effectively tackle the challenges inherent in cross-dataset transfer learning because of the distribution shifts between different datasets, thereby providing experimental support for the subsequent transfer of the model to other datasets. Based on the results from these three experimental groups, the subsequent experiments designed in this study adopted a dataset partitioning strategy that utilizes the REFLACX dataset for fine-tuning and the EGD dataset for validation.

#### 4.3.4. Ablasion Study

To validate the effectiveness of our proposed approach, we conducted a series of carefully designed ablation experiments to assess the individual and combined contributions of the feature fusion module, the inter- and intra-feature pool contrastive loss mechanisms, and the feature pool update strategy. The quantitative results of these component-wise evaluations are summarized in Table 3.

In this study, we systematically examined four critical components: the feature fusion module, align-contrastive loss, margin-contrastive loss, and the feature update mechanism. The results demonstrate that the model achieves its best classification performance when all four components are jointly incorporated.

Notably, when evaluating the label-specific contrastive loss module in isolation, we observed that integrating only the align-contrastive loss with the feature fusion module provided limited performance gains. In fact, this configuration resulted in declines in AUPRC and AUPRC compared to using the feature fusion module alone. This degradation may stem from the align-contrastive loss’s reliance on measuring similarity solely within the same feature pool. If the stored features lie near decision boundaries or in overlapping regions between pools, this intra-pool similarity constraint may introduce ambiguity and hinder effective learning. Substantial performance improvements were observed only when both the margin-contrastive loss and the feature pool update mechanism were introduced. These findings underscore the importance of their synergistic integration: while the margin-contrastive loss captures cross-pool feature relationships, the update strategy dynamically refines the feature distribution. Together, they effectively mitigate the limitations of isolated intra-pool constraints and enhance the model’s discriminative capability.

#### 4.3.5. Parameter Sensitivity Analysis

**Figure 7:**
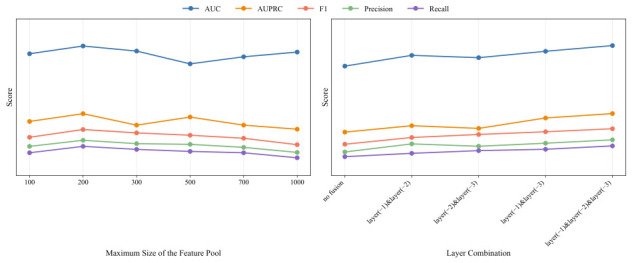
Experimental Results of Parameter Tuning for Maximum Feature Pool Size and Feature Fusion Layers.

Within the proposed model framework of this study, both the number of label-specific features stored in the feature pool and the number of network layers involved in the feature fusion module represent two parameters that warrant further in-depth investigation. We designed a parameter sensitivity analysis to identify the optimal experimental parameters that enable the model to achieve peak performance, with the specific experimental results illustrated in Figure 7. Our experimental configurations were as follows. When validating the parameter of maximum feature vector capacity in the feature pool, we set this parameter to 100, 200, 300, 500, 700, and 1000 respectively while keeping other experimental conditions constant, then evaluated the results across five performance metrics including AUC. For validating the feature fusion module, we conducted five experimental groups to examine configurations with no feature fusion, two-layer fusion, and three-layer fusion, where layer(−1), layer(−2), and layer(−3) denote the last, second-to-last, and third-to-last convolutional layers of the model, respectively. The experimental results demonstrate that the model achieved optimal performance with a feature vector count of 200 and a three-layer convolutional fusion scheme.

#### 4.3.6. Visualization of Feature Distribution

**Figure 8:**
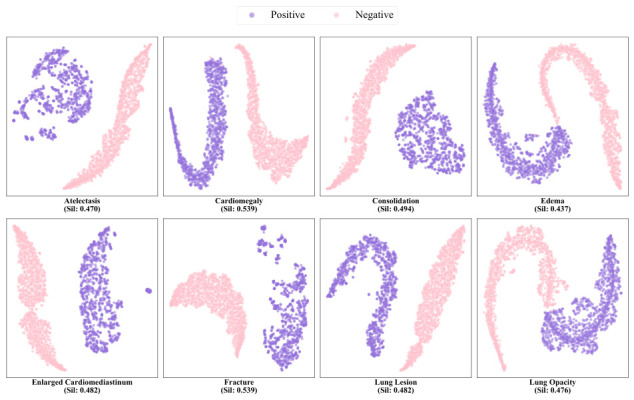
Visualization of Positive and Negative Feature Distributions for Representative Labels. The t-SNE plots illustrate the local cluster formations, while the accompanied Silhouette Scores provide quantitative validation of the intra-class cohesion and inter-class separability in the high-dimensional feature space.

To evaluate the effectiveness and discriminative power of the proposed Label-Specific Feature Extractor (LSFE) and the feature pool structure, we conducted t-SNE visualization experiments on the updated feature vectors following the training phase. The distribution of negative and positive features for representative labels is illustrated in the Figure 8. As observed from the visualization, the model successfully extracts highly representative feature vectors for each label through the proposed feature extraction and dynamic update mechanisms. Furthermore, a clear separation is maintained between the positive and negative feature clusters, providing empirical evidence for the robust classification performance of our framework across diverse thoracic diseases. While t-SNE effectively visualizes local cluster formations, it can sometimes distort global geometric relationships. To quantitatively validate the spatial separability of the extracted features, we calculated the Silhouette scores for the representative labels. The positive Silhouette scores objectively mathematically confirm that the proposed contrastive constraints successfully enforce compactness within the same class and dispersion across different classes. This confirms that the observed visual separation reflects a genuine and robust discriminative boundary in the original feature manifold.

## 5. Discussion

In this study, we propose a contrastive loss strategy based on label-specific features to constrain the feature extraction process, aiming to obtain more discriminative and representative label-aware embeddings. Comparative experiments with existing methods, along with ablation studies, demonstrate the effectiveness of the proposed approach. The resulting model achieves improved overall classification performance, particularly in the recognition of rare disease categories.

Nevertheless, several limitations in this study must be clearly acknowledged, which also pave the way for our future research endeavors.

First, due to computational resource constraints, the pre-training phase was conducted on a restricted subset of posteroanterior (PA) chest radiographs rather than the entirety of the large-scale datasets. This limitation in data scale may inherently restrict the diversity of the feature pool, potentially introducing distribution biases and limiting the model’s generalization capabilities across more diverse, multi-center patient populations. Future research will leverage enhanced high-performance computing resources to validate the framework on complete, full-scale datasets.

Second, while our ablation studies demonstrated the efficacy of the combined contrastive loss, relying solely on the align-contrastive loss yielded limited improvements. The synergistic dependency between the margin-contrastive loss, align-contrastive loss, and the dynamic update mechanism increases the overall complexity of the training pipeline and introduces hyperparameter sensitivity that requires careful tuning. Furthermore, although the Label-Specific Feature Extractor (LSFE) significantly improves quantitative metrics, its underlying decision-making process remains somewhat opaque, warranting further investigation into the clinical interpretability of the extracted feature vectors.

Finally, the current framework relies exclusively on 2D chest X-ray imaging. In real-world clinical workflows, physicians synthesize information from multiple sources. Future work will focus on integrating multimodal data to construct comprehensive patient profiles. For instance, fusing imaging features with unstructured clinical data, such as radiological reports that capture the visual attention patterns of radiologists, holds tremendous potential to further refine label-specific modeling and elevate overall diagnostic accuracy.

## Figures and Tables

**Table 1:** Quantitative comparison of our proposed framework with State-of-the-Art Models. Best results are highlighted in bold. Values are presented as Mean ± Std (%).

	AUC	AUPRC	F1-Score	Precision	Recall
ResNet50 + ImageNet [38]	0.7120 ± 0.0012	0.2049 ± 0.0035	0.2441 ± 0.0018	0.3047 ± 0.0042	0.2036 ± 0.0021
ResNet50 + CheXPter [28]	0.7212 ± 0.0019	0.2122 ± 0.0027	0.2503 ± 0.0027	0.2967 ± 0.0005	0.2165 ± 0.0041
CheXNet [12]	0.7377 ± 0.0023	0.2127 ± 0.0045	0.2712 ± 0.0015	0.3440 ± 0.0038	0.2239 ± 0.0013
SwinChex [32]	0.7329 ± 0.0031	0.2203 ± 0.0011	0.2846 ± 0.0032	0.3604 ± 0.0029	0.2351 ± 0.0033
CvTGNet [33]	0.7481 ± 0.0007	0.2313 ± 0.0025	0.2997 ± 0.0029	0.3471 ± 0.0017	0.2637 ± 0.0040
MLGCN [34]	0.7663 ± 0.0013	0.2356 ± 0.0049	0.2816 ± 0.0017	0.3096 ± 0.0003	0.2583 ± 0.0029
DANN [35]	0.7503 ± 0.0028	0.2557 ± 0.0014	0.2773 ± 0.0011	0.3174 ± 0.0024	0.2462 ± 0.0013
SimCLR [36]	0.7922 ± 0.0034	0.2580 ± 0.0006	0.2691 ± 0.0025	0.3197 ± 0.0040	0.2323 ± 0.0029
Class-Center [37]	0.8118 ± 0.0037	0.2645 ± 0.0016	0.3002 ± 0.0023	0.3738 ± 0.0002	0.2508 ± 0.0032
Ours	**0.8296 ± 0.0004**	**0.2969 ± 0.0020**	**0.3301 ± 0.0016**	**0.3943 ± 0.0046**	**0.2839 ± 0.0014**

**Table 2:** Comparison of Three Fine-Tuning and Validation Dataset Partitioning Schemes. Best results are highlighted in bold. Values are presented as Mean ± Std (%).

Fine-Tuning	Validation	AUC	AUPRC	F1-Score	Precision	Recall
REFLACX	EGD	**0.8296 ± 0.0004**	0.2969 ± 0.0020	**0.3301 ± 0.0016**	**0.3943 ± 0.0046**	**0.2839 ± 0.0014**
EGD	REFLACX	0.8284 ± 0.0019	**0.3011 ± 0.0027**	0.3194 ± 0.0028	0.3845 ± 0.0005	0.2732 ± 0.0039
Merged	Merged	0.8241 ± 0.0023	0.2997 ± 0.0045	0.3264 ± 0.0011	0.3906 ± 0.0038	0.2804 ± 0.0011

**Table 3:** Ablation Study of Proposed Modules.Best results are highlighted in bold. Values are presented as Mean ± Std (%).

Feature Fusion	Algin Loss	Margin Loss	Feature Update	AUC	AUPRC	F1-Score	Precision	Recall
✗	✗	✗	✗	0.8048 ± 0.0011	0.2630 ± 0.0032	0.3011 ± 0.0024	0.3715 ± 0.0041	0.2531 ± 0.0023
✓	✗	✗	✗	0.8292 ± 0.0018	0.2920 ± 0.0025	0.3129 ± 0.0017	0.3821 ± 0.0004	0.2654 ± 0.0026
✓	✓	✗	✗	0.8148 ± 0.0022	0.2861 ± 0.0045	0.3221 ± 0.0025	0.3840 ± 0.0037	0.2774 ± 0.0015
✓	✓	✓	✗	0.8254 ± 0.0030	0.2918 ± 0.0012	0.3223 ± 0.0027	0.3813 ± 0.0023	0.2791 ± 0.0035
✓	✓	✓	✓	**0.8296 ± 0.0004**	**0.2969 ± 0.0020**	**0.3301 ± 0.0016**	**0.3943 ± 0.0046**	**0.2839 ± 0.0014**

## Data Availability

Publicly available datasets were analyzed in this study. This data can be found here: [CheXpert: https://aimi.stanford.edu/datasets/chexpert-chest-x-rays] [REFLACX: https://physionet.org/content/reflacx-xray-localization/1.0.0/] [EGD: https://physionet.org/content/egd-cxr/1.0.0/], accessed on 6 April 2026.

## Abbreviations

The following abbreviations are used in this manuscript:

CXR	Chest X-ray
AUC	Area Under the Curve
AUPRC	Area Under the Precision-Recall Curve, AUPRC
CAD	Computer-Aided Diagnosis
DL	Deep Learning
LSFE	Label-Specific Feature Extractor
